# A numerical study on the dynamics of SIR epidemic model through Genocchi wavelet collocation method

**DOI:** 10.1038/s41598-025-93820-w

**Published:** 2025-03-21

**Authors:** Darshan Kumar Chiranahalli Vijaya, Prakasha Doddabhadrappla Gowda, Balachandra Hadimani

**Affiliations:** 1 Department of Mathematics, Davangere University, Shivagangotri, Davangere, 577007 India; 2 Department of Mathematics, Manipal Academy of Higher Education, Manipal Institute of Technology, Manipal, 576104 India

**Keywords:** Fractional differential equation, Genocchi wavelets, Epidemic model, Caputo fractional derivative, Diseases, Mathematics and computing

## Abstract

Epidemic models can play a major role in understanding the spread of diseases and their control. These mathematical models have plenty of significance in various scientific domains, including public health, to investigate disease propagation and ecology. This article explains the dynamics of SIR epidemic model of arbitrary order with aid of a precise numerical approach called Genocchi wavelet collocation method. The main purpose of this investigation is to explore and discover the results for system of nonlinear ordinary differential equations arising in the considered mathematical model and to investigate the dynamical aspects of SIR model via Caputo fractional derivative which is non-local in behaviour. The projected method depicts rapid algorithms and is extremely precise, reliable, and uses fewer computational resources. Also, this method is simpler than the other traditional numerical methods as it merges the operational matrix with the collocation method in order to transform fractional-order problem into algebraic equations which enables to obtain satisfactory results. The approximate solution obtained using proposed algorithm exposes the nature of their interactions. Furthermore, the numerical outcomes are represented through graphs for different fractional order and compared the results with Runge–Kutta method and residual power series method. The projected technique is very effective, accurate, free from controlling parameters and consume less time to investigate nonlinear complications arising in diverse fields of epidemical and biological models. Ultimately, the current study help to inspect the wild class of models and their performance which are occurring in real world.

## Introduction

Epidemiology is one of the branch of biology that deals with investigating the frequency of cases and problems which arises related to health in specific areas or groups. In a given population, an epidemic is the fast transmission of a viral disease to a immense number of individuals over short period of time. This transmission arises when host resistance to a freshly circulating unique vector or an old pathogen is abruptly decreased. It strives to offer statistics for the planning, implementation, and assessment of disease prevention, control, and treatment services by establishing service priorities. It helps to investigate the emergence and decline of the disease in the population in a short period, community diagnosis, assessment and planning, analysis of individual’s risks and possibilities of illness, syndrome recognition, and completion of the natural development of the illness or disease. Mathematical modelling is the act of defining real-world situations in terms of mathematical equations and also helps to understand the original problem, making predictions to uncover new features of real-world problems. Understanding disease transmission mechanisms is essential for controlling its spread in this real world. There are numerous articles on mathematical estimation in epidemiology. Ordinary differential equations with suitable parameters have been used to represent several real-world challenges^1–3^. Kermack and McKendrick^4^ demonstrated a epidemic model in order to better explain the characteristics of infectious disease in a homogeneous population. Ravichandran et al. introduced computer virus epidemic model and explored its behaviour with optimal control analysis to protect data and prevent viruses using Atangana–Baleanu (AB) fractional operator^5^. The dynamics of Zika virus model using AB fractional operator via *q*-homotopy analysis transform method has been studied by Veeresha et al.^6^. To learn more about the outbreak of epidemic diseases, their history, and strategies for tackling them using different analytical and numerical methods can be seen in the following cited references^7–18^.

Several challenges and weaknesses encountered when tackling memory and hereditary issues in classical calculus. To overcome this, *Leibniz* and *L’Hospital* proposed arbitrary derivatives and integrals leading to the development of fractional calculus (FC). FC is a mathematical discipline that defines derivatives and integrals of fractional order. The primary benefit of FC is that it provides an accurate representation of a wide range of nonlinear occurrences in this real-world. Moreover, it provides a solution in between intervals, allowing us to analyze the data more clearly. There are modified fractional operators, such as Riemann–Liouville fractional integral (RL), Hadamard fractional derivative, Grunwald–Letnikov derivative, Caputo fractional operator and so on^19–23^. The Caputo fractional derivative better captures the non-local transmission dynamics and aids to contain the historical effects od solution which leads to more accurate predictions of disease dynamics. It is well-suited for initial value problems and gives smoother behaviour compared to some other fractional derivative such as Riemann–Liouville fractional derivative. This smoothness property is desirable as it yields more continuous and physically realistic models. Moreover, It incorporates memory effects and advances in gaining the accurate results of epidemic models, especially in capturing delayed, complex, or non-local dynamics. It is useful in super diffusion models where disease outbreak spreads rapidly in highly populated areas. Fractional differential equations (FDE) have vast range of popularity and prominence because of their broad spectrum of significance in science and engineering. FDE’s are rapidly being used to deal with problems arising in physics, ecology, epidemics, engineering, diseases, hydrodynamics, biology, and other physical phenomena^24–32^.

Wavelets are a family of functions composed of translation and dilation of only one function called the mother wavelet. This wavelet theory has been greatly refined by the investigation of Morlet, Stromberg, Meyer, Grossmann, and Daubechies. Its unique features includes compact support, orthogonality, and multiresolution analysis attract a lot of research attention. Ramzan et al.^33^ presented Nipah Virus model considering the fractal-fractional operator in the sense of Mittag-leffler kernel using the Lagrange interpolation polynomial technique. Using Newton polynomials, secret sharing for multi-factor authentication had been discussed by Bezzateev et al.^34^. Gandha and Santoso^35^ implemented Automatic Model Generation method using newton polynomials in the ultrasonic sensor for calibration process. Lagrange interpolation polynomial technique had been utilized by Rashid et al.^36^ to study cholera infectious dynamics with the help of fractal-fractional derivative. With the aid of Caputo derivative, Kumar and Gupta^37^ derived lagrange polynomial operational matrices to solve non-linear Volterra-Fredholm integro-differential equations. A number of wavelet based numerical techniques have been effectively resolved in the following literature: Fibonacci wavelet method, Taylor wavelet collocation approach, Chebyshev wavelet method, Laguerre wavelet approach, Bernoulli wavelet scheme, Haar wavelet collocation method and so on^38–43^.

Genocchi wavelets are one of the types of continuous wavelets. The considered model can be reduced to a series of algebraic equations with the aid of operational matrices to estimate the integrals. The innovation of the Genocchi wavelet collocation method (GWCM) is as follows: The polynomials in Genocchi wavelets $\psi _m(\tau )$ have less coefficients than Legendre polynomials $L_m(\tau )$. Comparable terms in Legendre polynomials $L_m(\tau )$ have a larger coefficient than each terms in Genocchi polynomials $\psi _m(\tau )$. Moreover, the presented approach reduce computational errors and it can cut down on CPU usage. Sharp edge/jump discontinuities are well suitable for solutions using the Genocchi wavelet collocation approach. It can be employed to determine the numerical solution of an ordinary differential system of higher order by making minor modifications to the procedure. This method can be investigated for other mathematical disciplines with distinct physical and biological circumstances including PDEs. Without adjusting any parameters, this approach enables the direct solution for differential equations of fractional order, differential equations with delay and stiff systems. GWCM depicts rapid algorithms and is extremely precise and it is more direct, reliable, and uses fewer computational resources than Legendre, Chebyshev, and Bernoulli wavelets. We discovered the following renowned articles on solving mathematical problems with Genocchi Wavelets. Dehestani et al.^44^ implemented GWCM to obtain numerical solution for delay fractional differential equations with the aid of Caputo fractional derivative. The GWCM has applied to solve variable-order partial differential equations by Kanwal et al.^45^. Rahimkhani and Ordokhani^46^ used Genocchi wavelets to solve various kinds of differential equations through artificial neural network method and least squares support vector regression method. In 2021, the time fractional Rosenau-Hyman equation is solved using Genocchi wavelet method using Maple software by Cinar et al.^47^ In this paper, we study SIR influenza model through GWCM. Many authors have gathered attention towards Genocchi wavelets and solved various fractional differential equations using this method in their research works^48–52^. This paper is organised in the following manner: Sect. “Preliminaries” comprises of basic definitions of fractional calculus and Genocchi wavelets. The considered short-termed influenza epidemical model has been described in Sect. “Model description”. Section “Genocchi wavelet matrix of integration (GWMI)” covers the Genocchi wavelet basis and matrix of integration. The projected algorithm of Genocchi wavelets for the considered SIR model has been described in Sect. “Genocchi wavelet collocation method”. In Sect. “Results and discussion”, numerical investigation through graphs and solutions obtained using GWCM has been explained. Finally, the conclusion of the article has been clarified in Sect. “Conclusion”.

## Preliminaries

In this part, We briefly explain some essential definitions related to fractional calculus and Genocchi Wavelets.

### Definition 1

The Riemann–Liouville fractional integral of order $\gamma$ for the function $f\left( \tau \right) \in C_\mu \left( \mu \ge -1\right)$,^19,20^ is stated as

1 $$\begin{aligned} J^\gamma f\left( \tau \right) =\frac{1}{\mathrm {\Gamma }\left( \gamma \right) }\int _{0}^{\tau }{\left( \tau -\vartheta \right) ^{\gamma -1}f\left( \vartheta \right) d\vartheta }. \end{aligned}$$

### Definition 2

For the function $\mathcal {V}(t)\in C_{-1}^r$, the Caputo fractional derivative^20,21^ of order $\gamma$ is defined as

2 $$_{0} {\mathcal{D}}_{\tau }^{\gamma } {\mathcal{V}}\left( t \right) = {\text{ }}\left\{ {\begin{array}{*{20}l} {\frac{{d^{r} {\mathcal{V}}\left( t \right)}}{{d\tau ^{r} }},} \hfill & {\gamma = r \in \mathbb{N},} \hfill \\ {\frac{1}{{\Gamma (r - \gamma )}}\int_{0}^{t} {\left( {\tau - \theta } \right)^{{r - \gamma - 1}} {\mathcal{V}}^{{(r)}} \left( \theta \right)d\theta ,\gamma \in (r - 1,r),} } \hfill & {r \in \mathbb{N}.} \hfill \\ \end{array} } \right.$$

The notation $\mathbb {N}$ denotes the set of natural numbers.

### Definition 3

(Genocchi wavelets) The Genocchi wavelets in the interval [0, 1) are defined^48,52^ as follows:

3 $$\psi _{{n,m}} (\tau ) = \left\{ {\begin{array}{*{20}l} {2^{{\frac{{k - 1}}{2}}} \tilde{G}_{m} (2^{k} \tau - \tilde{n}),} \hfill & {\frac{{\tilde{n}}}{{2^{{k - 1}} }} \le \tau \le \frac{{\tilde{n} + 1}}{{2^{{k - 1}} }},} \hfill \\ {0,} \hfill & {Otherwise,} \hfill \\ \end{array} } \right.$$

with

$$\tilde{G}_{m} (2^{k} \tau - n + 1) = \left\{ {\begin{array}{*{20}l} {1,} \hfill & {m = 1,} \hfill \\ {\frac{1}{{\sqrt {\frac{{2( - 1)^{{m - 1}} (m!)^{2} }}{{(2m)!}}g_{{2m}} } }}G_{m} (2^{k} \tau - n + 1),} \hfill & {m > 1,} \hfill \\ \end{array} } \right.$$

where $m=0,1,2,\ldots ,M-1,n=1,2,\ldots ,2^{k-1}.$ *k* is a positive integer and *m* is the order of Genocchi polynomial.

### Remark 1

The error analysis of proposed Genocchi wavelets are examined in^49,50^ Let $f(t) \in C^{n+1}[0,1], Y = Span\{G_1(t),G_2(t),\ldots ,G_N(t)\}$ and the approximation of *f*(*t*) in *Y* is $C^T G(T)$, then

$$\begin{aligned} ||f(t)-C^T G(t)|| \le \frac{h^\frac{2n+3}{2} R}{(n+1)!\sqrt{2n+3}}, \ \ \ t\in [t_i,t_{i+1}] \subseteq [0,1], \end{aligned}$$

where $h=t_{i+1}-t_i$, $i=1,2,\ldots ,n$ and $R=max_{t\in [t_i,t_{i+1}]} |f^{(n+1)}(t)|$, Thus *f*(*t*) has error on the interval [0, 1].

## Model description

We consider the short-termed influenza epidemic model^7^ and we make the following assumptions:

- The population should be large and closed, that means during this epidemic, there is no birth, death, immigration, and emigration of individuals,
- Recovered individuals may infected again, and
- The parameters are fixed and do not vary.

**Fig. 1:**
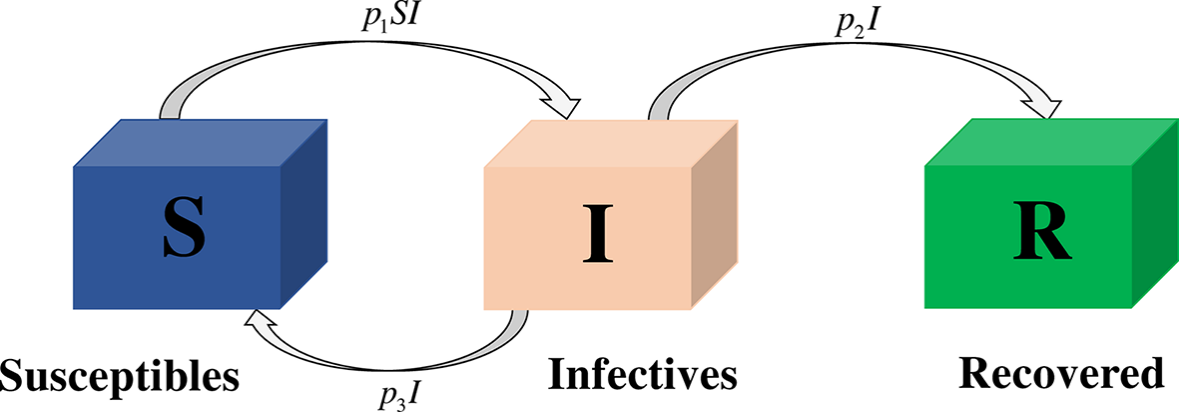
SIR epidemic model.

In a particular region, the total population *N* is divided into three distinct compartments: *S*(*t*) signifies the susceptible people, *I*(*t*) represents the number of infected individuals, and *R*(*t*) symbolizes recovered individuals in time *t*. Permitting susceptible individuals to get infected with the disease by interacting with infected people at an infection rate of $p_1$, and a person with the infection to suffer from the disease encounters recovered and susceptible individuals at rates of $p_2$ and $p_3$, respectively. The classical SIR epidemic model of short-termed influenza is illustrated in Fig. 1 and it is demarcated as

4 $$\begin{aligned} \frac{d S(\tau )}{d\tau }= & -p_1 S(\tau )I(\tau )+p_3I(\tau ),\nonumber \\ \frac{d I(\tau )}{d\tau }= & p_1 S(\tau )I(\tau )-p_3I(\tau )-p_2I(\tau ),\nonumber \\ \frac{d R(\tau )}{d\tau }= & p_2I(\tau ). \end{aligned}$$

The above epidemic model^7^ of fractional order is examined using Caputo operator is demarcated as follows,

5 $$\begin{aligned} \mathcal {D}_0^\gamma S(\tau )= & -p_1 S(\tau )I(\tau )+p_3I(\tau ),\nonumber \\ \mathcal {D}_0^\gamma I(\tau )= & p_1 S(\tau )I(\tau )-p_3I(\tau )-p_2I(\tau ),\nonumber \\ \mathcal {D}_0^\gamma R(\tau )= & p_2I(\tau ). \end{aligned}$$

where $0<\gamma \le 1$ be the order of the fractional operator. The positive constants $p_1, p_2,$ and $p_3$ denote the infection, removal, and recovery rates, respectively. The initial assumptions for the considered epidemic model is given as

6 $$\begin{aligned} S(0)=S_0,\ \ I(0)=I_0,\ \ R(0)=R_0. \end{aligned}$$

## Genocchi wavelet matrix of integration (GWMI)

The Genocchi wavelet basis when $k = 1$ and $M = 6$ is defined as follows^52^:

$$\begin{aligned} \psi _{1,0}(\tau )= & 1,\\ \psi _{1,1}(\tau )= & \sqrt{3}(-1+2\tau ),\\ \psi _{1,2}(\tau )= & \sqrt{30}(-1+\tau )\tau ,\\ \psi _{1,3}(\tau )= & \sqrt{\frac{35}{17}}(1-6\tau ^2+4\tau ^3),\\ \psi _{1,4}(\tau )= & 3\sqrt{\frac{70}{31}}(\tau -2\tau ^3+\tau ^4),\\ \psi _{1,5}(\tau )= & 3\sqrt{\frac{154}{691}}(-1+2\tau )(1+\tau -\tau ^2)^2.\\ \psi _{1,6}(\tau )= & 2\sqrt{\frac{3003}{5461}}\tau (-3+5\tau ^2-3\tau ^4+\tau ^5).\\ \psi _{1,7}(\tau )= & 3\sqrt{\frac{715}{929569}}(17-84\tau ^2+70\tau ^4-28\tau ^6+8\tau ^7). \end{aligned}$$

Now, integrating the first six wavelet bases concerning $\tau$ from limits 0 to $\tau ,$ the linear combination of Genocchi wavelet bases can be expressed in following manner:

$$\begin{aligned} &\int\limits_{0}^{\tau } {\psi _{{1,0}} } (\tau )d\tau = {\text{ }}\left[ {\frac{1}{2}{\mkern 1mu} {\mkern 1mu} \frac{1}{{2\sqrt 3 }}{\mkern 1mu} {\mkern 1mu} 0{\mkern 1mu} {\mkern 1mu} 0{\mkern 1mu} {\mkern 1mu} 0{\mkern 1mu} {\mkern 1mu} 0} \right]\psi _{6} (\tau )\\ &\int\limits_{0}^{\tau } {\psi _{{1,1}} } (\tau )d\tau = \left[ {0{\mkern 1mu} 0{\mkern 1mu} {\mkern 1mu} \frac{1}{{\sqrt {10} }}{\mkern 1mu} {\mkern 1mu} {\mkern 1mu} 0{\mkern 1mu} {\mkern 1mu} 0{\mkern 1mu} {\mkern 1mu} 0} \right]\psi _{6} (\tau )\\ & \int\limits_{0}^{\tau } {\psi _{{1,2}} } (\tau )d\tau = {\text{ }}\left[ { - \frac{{\sqrt {\frac{5}{6}} }}{2}{\mkern 1mu} {\mkern 1mu} 0{\mkern 1mu} 0{\mkern 1mu} {\mkern 1mu} \frac{{\sqrt {\frac{{17}}{{42}}} }}{2}{\mkern 1mu} {\mkern 1mu} 0{\mkern 1mu} {\mkern 1mu} 0} \right]\psi _{6} (\tau )\end{aligned}$$

$$\begin{aligned} &\int\limits_{0}^{\tau } {\psi _{{1,3}} } (\tau )d\tau = {\text{ }}\left[ {0{\mkern 1mu} {\mkern 1mu} 0{\mkern 1mu} {\mkern 1mu} 0{\mkern 1mu} {\mkern 1mu} 0{\mkern 1mu} {\mkern 1mu} \frac{{\sqrt {\frac{{31}}{{34}}} }}{3}{\mkern 1mu} {\mkern 1mu} 0} \right]\psi _{6} (\tau )\\& \int\limits_{0}^{\tau } {\psi _{{1,4}} } (\tau )d\tau = \left[ {3\sqrt {\frac{7}{{310}}} {\mkern 1mu} {\mkern 1mu} 0{\mkern 1mu} {\mkern 1mu} 0{\mkern 1mu} {\mkern 1mu} 0{\mkern 1mu} {\mkern 1mu} 0{\mkern 1mu} {\mkern 1mu} \frac{{\sqrt {\frac{{691}}{{1705}}} }}{2}} \right]\psi _{6} (\tau )\\ & \int\limits_{0}^{\tau } {\psi _{{1,4}} } (\tau )d\tau = {\text{ }}\left[ {0{\mkern 1mu} {\mkern 1mu} 0{\mkern 1mu} {\mkern 1mu} 0{\mkern 1mu} {\mkern 1mu} 0{\mkern 1mu} {\mkern 1mu} 0{\mkern 1mu} {\mkern 1mu} 0} \right]\psi _{6} (\tau ) \end{aligned}$$

7 $$\begin{aligned} \int \limits _0^\tau \psi (\tau ) d\tau = P_{6\times 6} \psi _6 (\tau ) + \bar{\psi }_6 (\tau ) \end{aligned}$$

where

$$\begin{aligned} \psi _{6} (\tau )= & \left[ \psi _{1,0} (\tau ),\psi _{1,1} (\tau ),\psi _{1,2} (\tau ),\psi _{1,3} (\tau ),\psi _{1,4} (\tau ),\psi _{1,5} (\tau )\right] ^{T} \\ B_{6 \times 6}= & \begin{bmatrix} \frac{1}{2} & \frac{1}{2\sqrt{3}} & 0 & 0 & 0 & 0 \\ 0 & 0 & \frac{1}{{\sqrt{10} }} & 0 & 0 & 0 \\ - \frac{{\sqrt{5/6} }}{2} & 0 & 0 & \frac{\sqrt{\frac{17}{42} }}{2} & 0 & 0 \\ 0 & 0 & 0 & 0 & \frac{{\sqrt{\frac{31}{34}} }}{2} & 0 \\ 3\sqrt{\frac{7}{310}} & 0 & 0 & 0 & 0 & \frac{\sqrt{\frac{691}{1705}}}{2} \\ 0 & 0 & 0 & 0 & 0 & 0 \end{bmatrix} \\ \bar{\psi }_{6} (\tau )= & \begin{bmatrix} 0\\ 0\\ 0\\ 0\\ 0\\ \sqrt{\frac{5461}{53898}}\psi _{1,6}(\tau ) \end{bmatrix}. \end{aligned}$$

The generalized form of *n*-wavelet basis of first integration at $k = 1$ is demarcated as:

$$\begin{aligned} \int \limits _0^\tau \psi (\tau ) d\tau = P_{n \times n} \psi (\tau ) + {\bar{\psi }}_n (\tau ). \end{aligned}$$

## Genocchi wavelet collocation method

This section explores the numerical approach to solve the ODEs that describes the fractional SIR epidemic model via GWCM^52^. Let

8 $$\begin{aligned} \frac{dS(\tau )}{d\tau }= & A^T H(\tau )\nonumber \\ \frac{dI(\tau )}{d\tau }= & B^T H(\tau )\nonumber \\ \frac{dR(\tau )}{d\tau }= & C^T H(\tau ) \end{aligned}$$

where

$$\begin{aligned} A^T= & \Big [a_{1,0},a_{1,1},\ldots a_{1,M-1},a_{2,0},a_{2,1},\ldots a_{2,M-1},\ldots , \ldots a_{2^{k-1},0},a_{2^{k-1},1},\ldots a_{2^{k-1},M-1}\Big ]\\ B^T= & \Big [b_{1,0},b_{1,1},\ldots b_{1,M-1},b_{2,0},b_{2,1},\ldots b_{2,M-1},\ldots , \ldots b_{2^{k-1},0},b_{2^{k-1},1},\ldots b_{2^{k-1},M-1}\Big ]\\ C^T= & \Big [c_{1,0},c_{1,1},\ldots c_{1,M-1},c_{2,0},c_{2,1},\ldots c_{2,M-1},\ldots , \ldots c_{2^{k-1},0},c_{2^{k-1},1},\ldots c_{2^{k-1},M-1}\Big ] \\ \psi (\tau )= & \left[ \psi (\tau )_{1,0},\ldots \psi (\tau )_{1,M-1},\psi (\tau )_{2,0},\ldots \psi (\tau )_{2,M-1},\ldots \psi (\tau )_{2^{k-1},0},\ldots \psi (\tau )_{2^{k-1},M-1} \right] . \end{aligned}$$

*A*, *B*,  and *C* are unknown coefficients which are to be determined and $\psi (\tau )$ is the Genocchi wavelets basis. Integrating the system in Eq. (8) with regard to variable $\tau$ from 0 to $\tau$ and interpreting the initial assumptions in terms of wavelet functions, we have

9 $$\begin{aligned} S(\tau )= & D^T\psi (\tau )+A^T\begin{bmatrix} P\psi (\tau )+ \bar{\psi } (\tau ) \end{bmatrix}\nonumber \\ I(\tau )= & E^T\psi (\tau )+B^T\begin{bmatrix} P\psi (\tau )+ \bar{\psi } (\tau ) \end{bmatrix}\nonumber \\ R(\tau )= & F^T\psi (\tau )+C^T\begin{bmatrix} P\psi (\tau )+ \bar{\psi } (\tau ) \end{bmatrix}. \end{aligned}$$

Here, *D*, *E*,  and *F* are known vectors. Now, differentiating Eq. (9) with regard to $\tau$ fractionally using Definition 2. We get $\mathcal {D}^\gamma S(\tau ),\ \mathcal {D}^\gamma I(\tau ),\ \mathcal {D}^\gamma R(\tau ),$ respectively.

10 $$\begin{aligned} \mathcal {D}^\gamma S(\tau )= & D^T\mathcal {D}^\gamma {\psi (\tau )}+A^T\mathcal {D}^\gamma {\begin{bmatrix} P\psi (\tau )+ \bar{\psi } (\tau ) \end{bmatrix}}\nonumber \\ \mathcal {D}^\gamma I(\tau )= & E^T\mathcal {D}^\gamma {\psi (\tau )}+B^T\mathcal {D}^\gamma {\begin{bmatrix} P\psi (\tau )+ \bar{\psi } (\tau ) \end{bmatrix}}\nonumber \\ \mathcal {D}^\gamma R(\tau )= & F^T\mathcal {D}^\gamma {\psi (\tau )}+C^T\mathcal {D}^\gamma {\begin{bmatrix} P\psi (\tau )+ \bar{\psi } (\tau ) \end{bmatrix}}. \end{aligned}$$

Substitute these fractional values obtained along with Eq. (9) in the model and collocating the system 5 with the following points $\tau _j=\frac{2j-1}{2^{k}M},\ j=1,2,\ldots M.$ Then, we have get the system of algebraic equations which are derived using collocation points and we extract the unknown coefficients *A*, *B* and *C* with the aid of Newton-Raphson method.

The unknown coefficients obtained using Newton-Raphson method for $\gamma =1$ are as follows

$$\begin{aligned} A^T= & \Big [-8.278493,-1.250779,-0.194440,-0.026018,-0.002154,\ \ \ 0.000415\Big ]\\ B^T= & \Big [\ \ \ \ 7.301170,\ \ 1.099036,\ \ 0.169430,\ \ \ 0.022155,\ \ 0.001655,\ -0.000443\Big ]\\ C^T= & \Big [\ \ \ \ 0.977323,\ \ 0.151743,\ \ 0.025011,\ \ \ 0.003863,\ \ 0.000499,\ \ \ 0.000027\Big ] \end{aligned}$$

Substituting these unknown coefficients obtained in the considered model Eq. (9), we estimate the numerical values for $S(\tau ), I(\tau )$ and $R(\tau )$ for the considered model (5). Here, the situation in which a small number of people or individuals is present and they are suffering from an infectious disease, which is retained in a entire population capable of being infected with constant values be $p_1=0.001, p_2=0.072,$ and $p_3=0.005$ which specifies the infection, removal, and recovery rates, respectively. Suppose the initial assumptions for the considered epidemic model as in^7^ is given as

11 $$\begin{aligned} S(0)=620,\ \ I(0)=10,\ \ R(0)=70. \end{aligned}.$$

So, the entire human population is $N=620+10+70=700.$

**Fig. 2:**
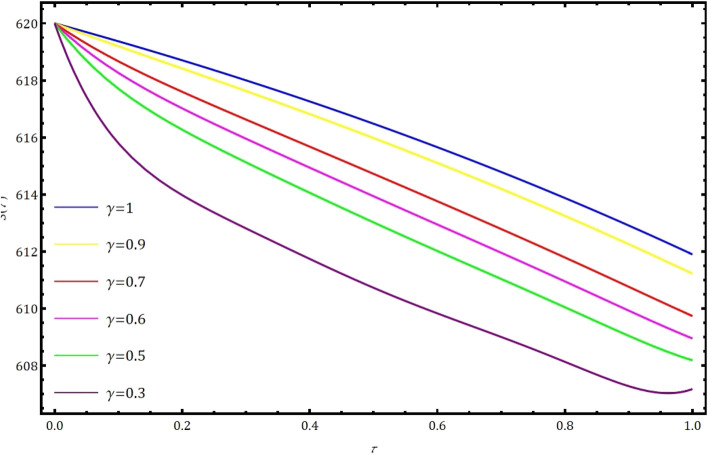
Nature of behavior of $S(\tau )$ for distinct values of $\gamma$ when $k=1$ and $M=6$ using GCWM.

**Fig. 3:**
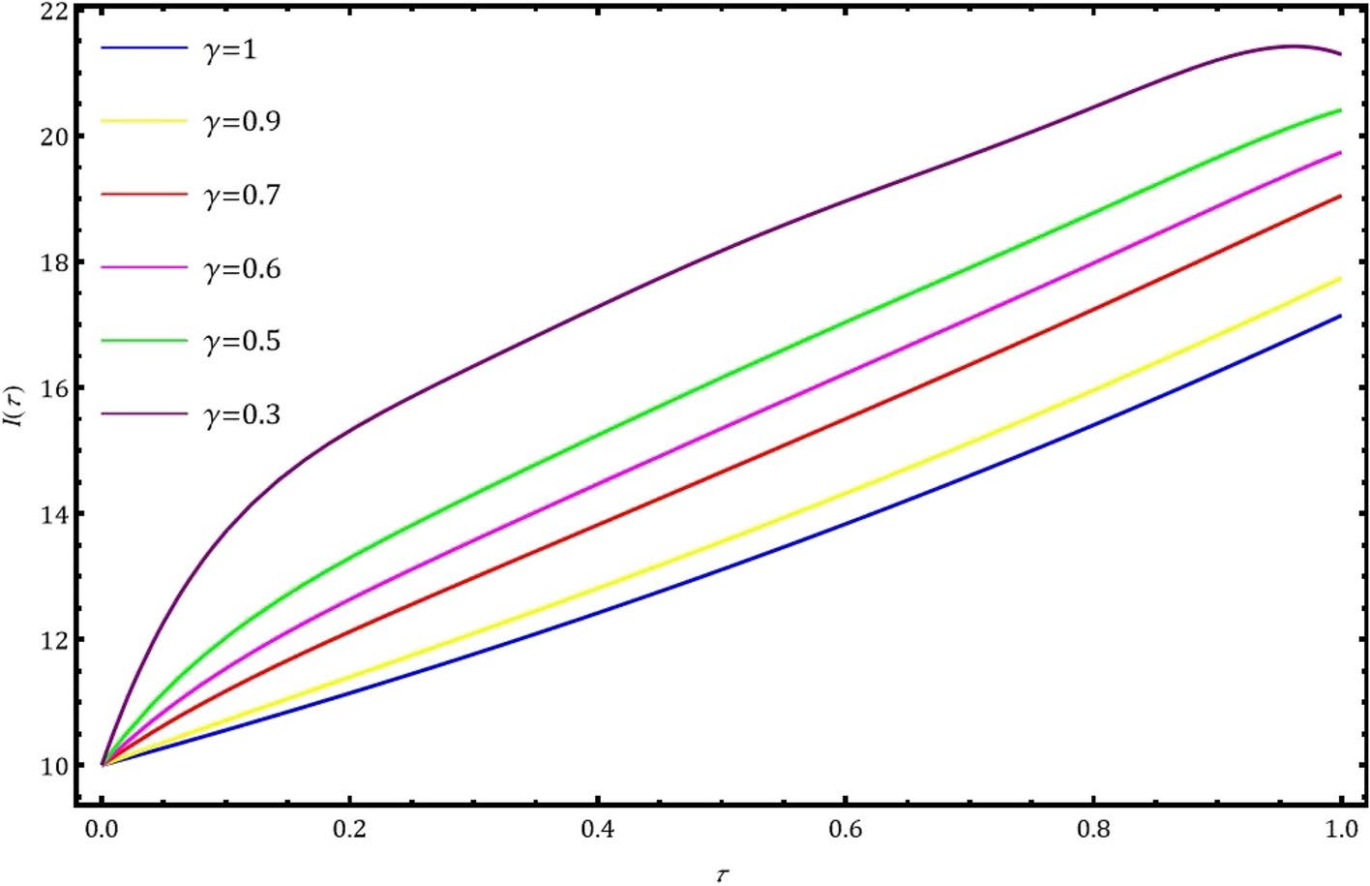
Nature of behavior of $I(\tau )$ for distinct values of $\gamma$ when $k=1$ and $M=6$ using GCWM.

**Fig. 4:**
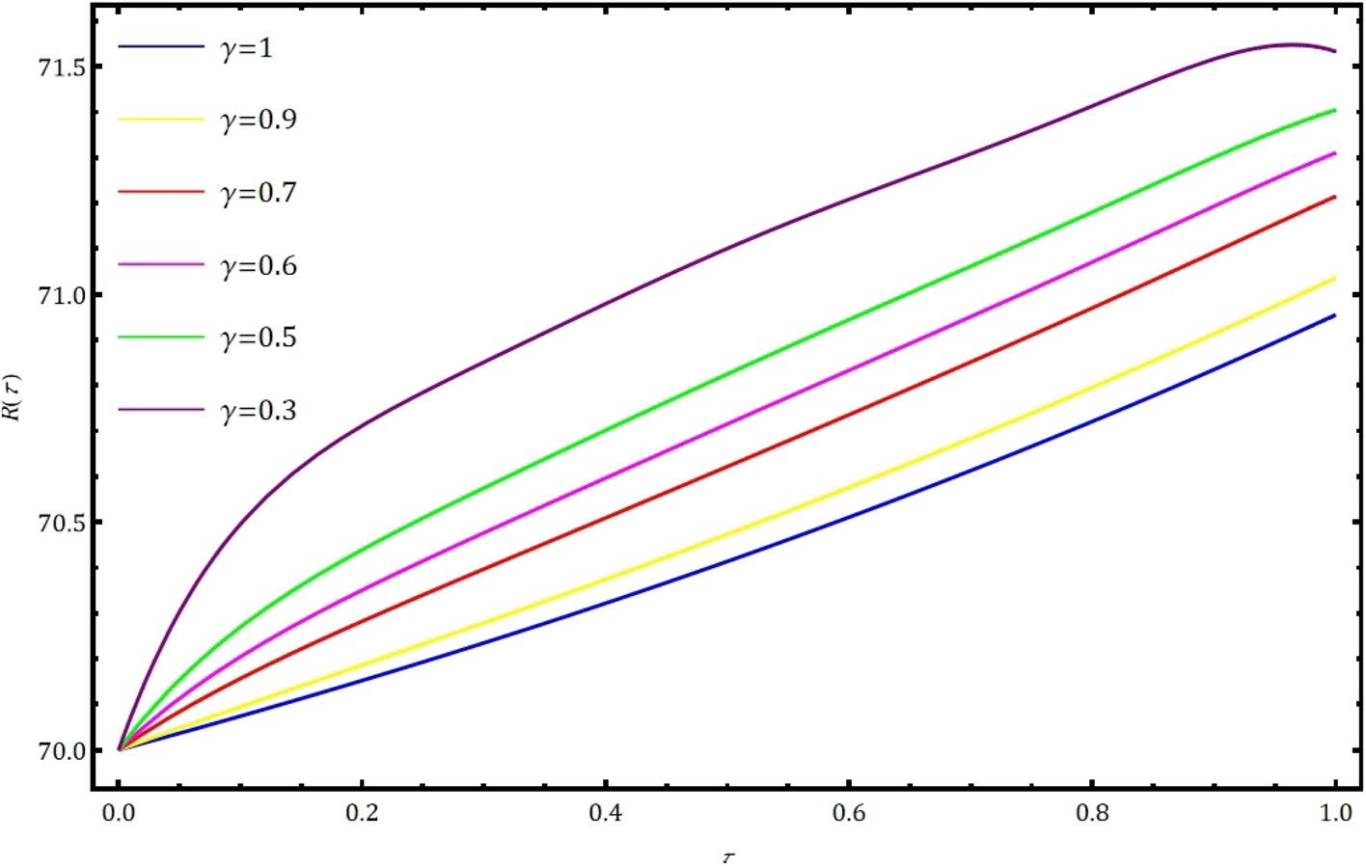
Nature of behavior of recovered individuals $R(\tau )$ for distinct values of $\gamma$ using GWCM.

**Fig. 5:**
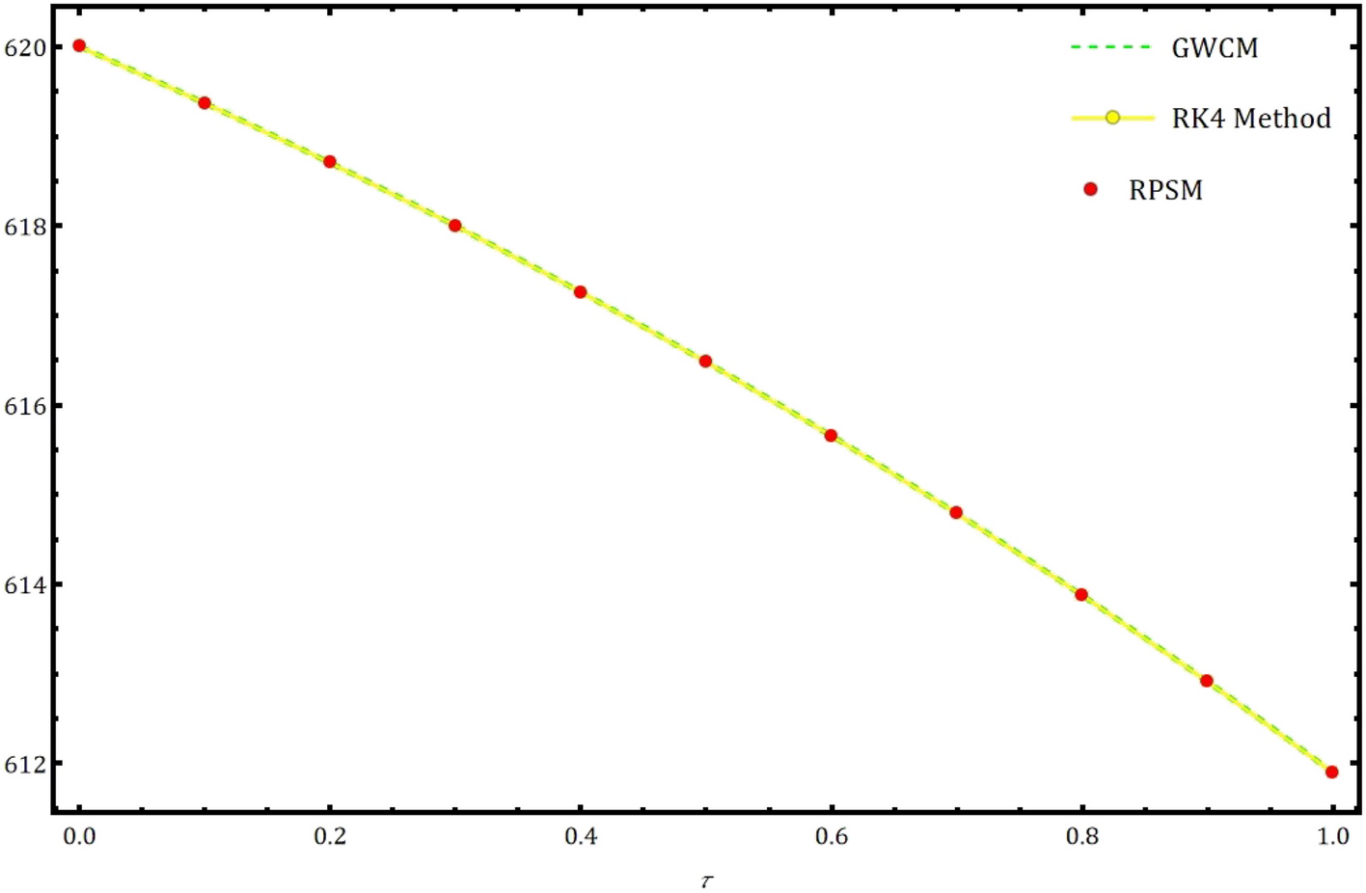
Achieved solution of $S(\tau )$ using GWCM compared with RK4 method and RPSM.

**Fig. 6:**
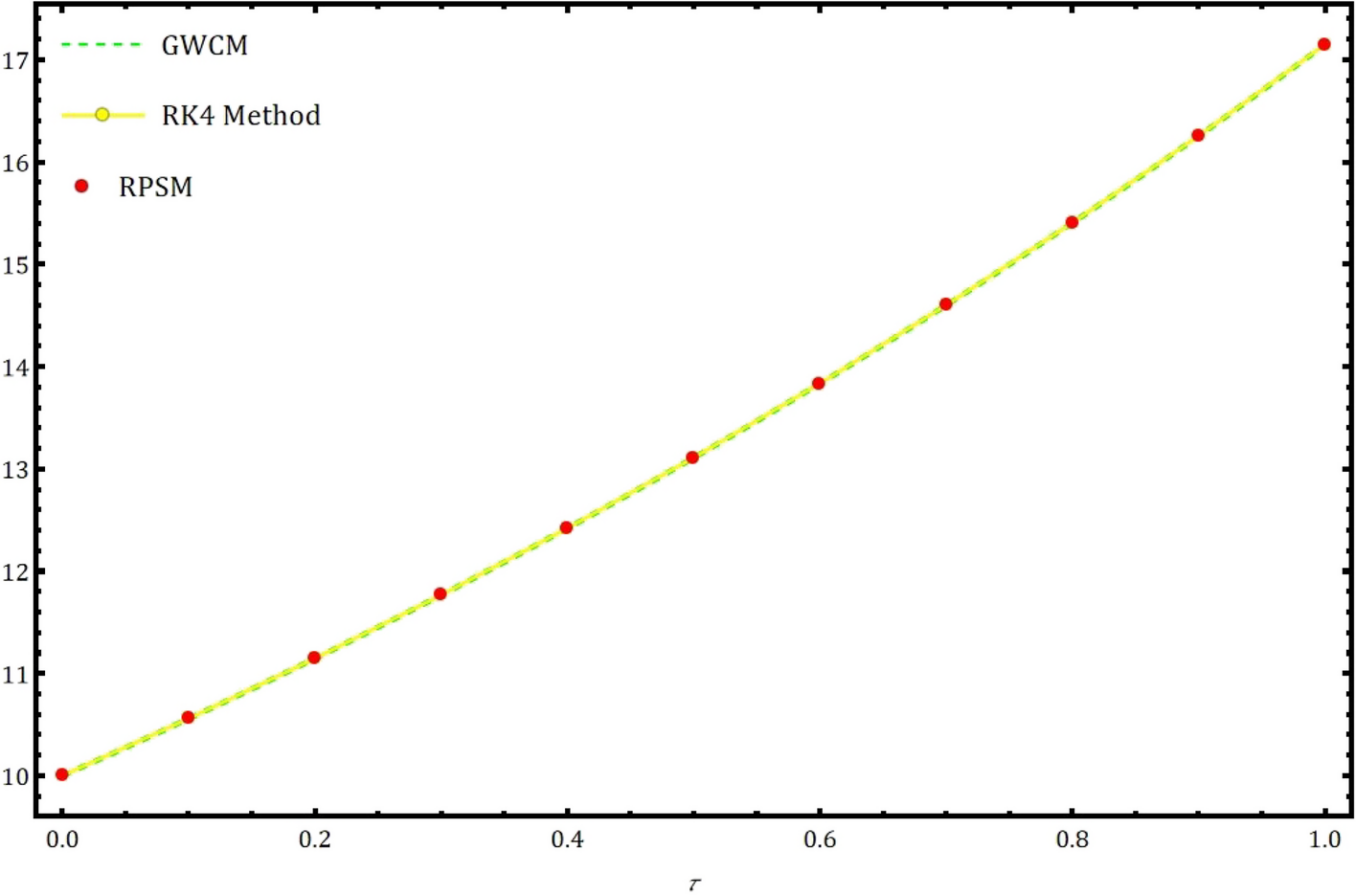
The approximate solution of $I(\tau )$ using GWCM in comparison with RK4 method and RPSM.

**Fig. 7:**
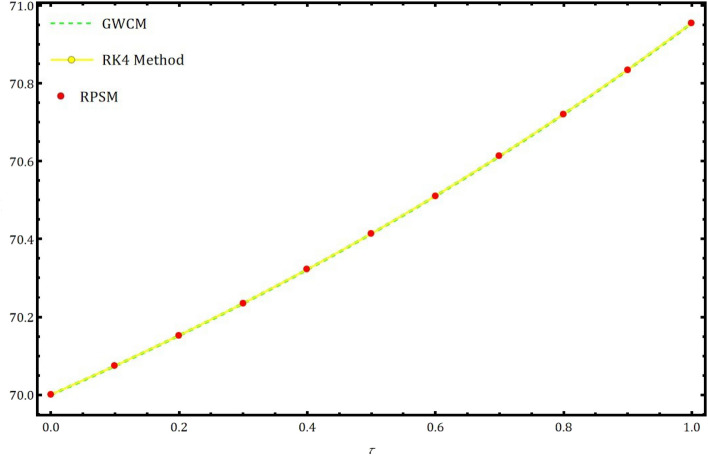
Approximate solution of $R(\tau )$ using the GWCM in comparison with RK4 method and RPSM.

**Fig. 8:**
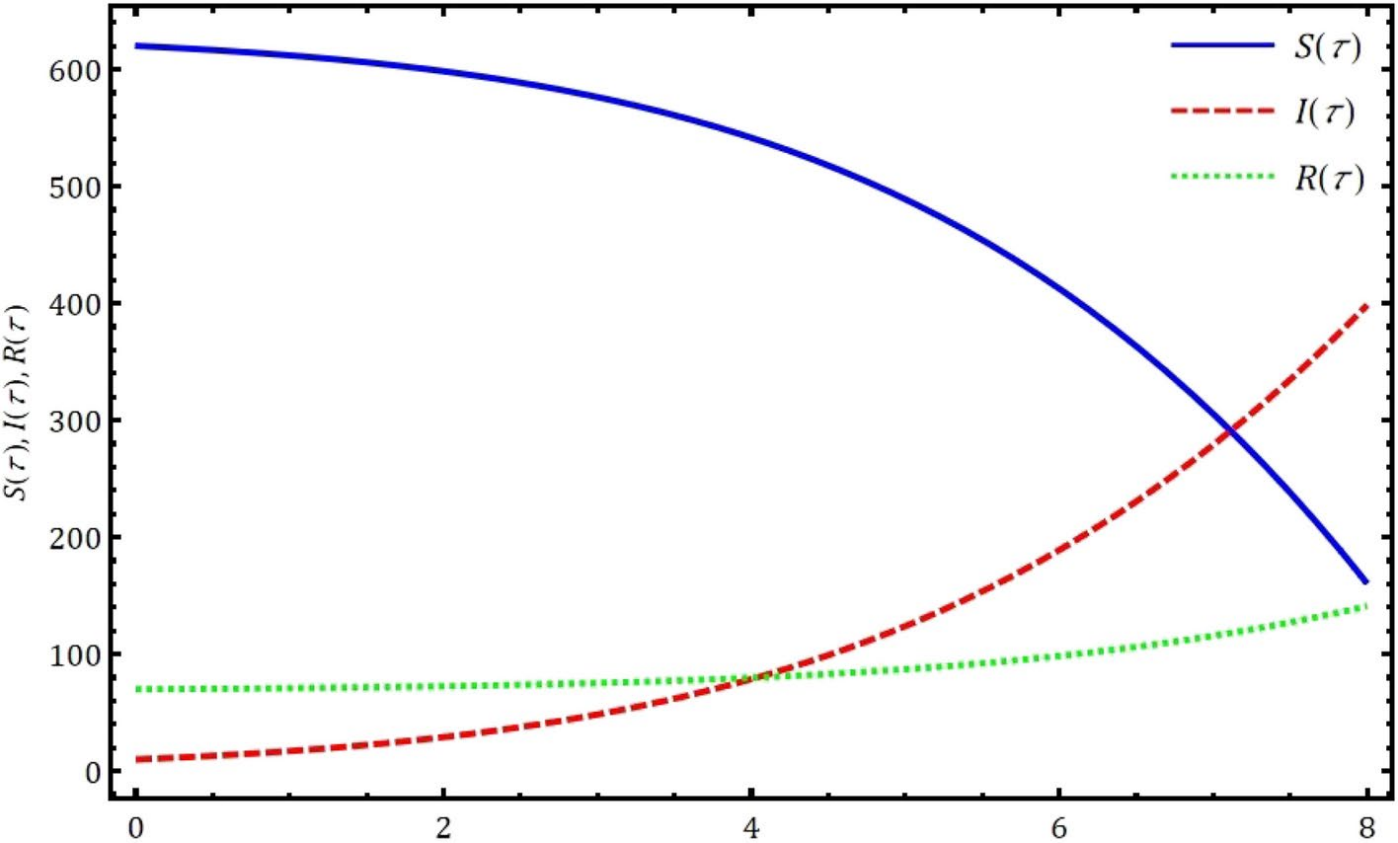
Approximate solution of SIR epidemic model 5 using the proposed GWCM at $\gamma =1.$

**Table 1:** The approximate values using RK4 method, RPSM and proposed GWCM for $S(\tau )$ of Eq. (5) at $\gamma =1.$

$\tau$	Solution using	Solution using	Solution using
	RK4 method	RPSM^7^	GWCM
0.00	620.000000000000000	620.000000000000000	620.0000000000000
0.10	619.368327701129830	619.368327657510350	619.3683276172103
0.20	618.702153816378770	618.702153724652930	618.7021536932915
0.30	617.999682039910570	617.999681895280220	617.9996818627100
0.40	617.259032603941480	617.259032401282640	617.2590323636210
0.50	616.478239561945540	616.478239295794650	616.4782421581235
0.60	615.655248125904110	615.655247790443130	615.6552477513076
0.70	614.787912080138200	614.787911669181200	614.7879116245700
0.80	613.873991297021010	613.873990803999960	613.8739907574077
0.90	612.911149382834760	612.911148800787370	612.9111487634786
1.00	611.896951485213090	611.896950806769950	611.8969507254561

**Table 2:** The approximate values using RK4 method, RPSM and proposed GWCM for infected individuals $I(\tau )$ for Eq. (5) at $\gamma =1.$

$\tau$	Solution using	Solution using	Solution using
	RK4 method	RPSM^7^	GWCM
0.00	10.000000000000000	10.000000000000000	10.000000000000000
0.10	10.557682415377140	10.557682454124135	10.557682492954838
0.20	11.145742665297318	11.145742746766953	11.145742776768072
0.30	11.765752718571312	11.765752847013921	11.765752878029145
0.40	12.419356430322715	12.419356610275818	12.419356646042534
0.50	13.108271733277171	13.108271969577906	13.108272005874188
0.60	13.834292759467655	13.834293057265034	13.834293094072999
0.70	14.599291870396474	14.599292235163372	14.599292277067361
0.80	14.599291870396474	15.405222008647254	15.405222052236754
0.90	16.254116280801693	16.254116797279011	16.254116831658386
1.00	17.148093929799238	17.148094531720652	17.148094608528900

**Table 3:** The approximate values using RK4 method, RPSM and proposed GWCM for $R(\tau )$ of Eq. (5) at $\gamma =1.$

$\tau$	Solution using	Solution using	Solution using
	RK4 method	RPSM^7^	GWCM
0.00	70.000000000000000	70.000000000000000	70.00000000000000
0.10	70.073989883493013	70.073989888365404	70.07398988983490
0.20	70.152103518323841	70.152103528579929	70.15210352994040
0.30	70.234565241518069	70.234565257705697	70.23456525926090
0.40	70.321610965735758	70.321610988441307	70.32161099033648
0.50	70.413488704777279	70.413488734627379	70.41348873673917
0.60	70.510459114628276	70.510459152291887	70.51045915461945
0.70	70.612796049465331	70.612796095655639	70.61279609836264
0.80	70.720787131876406	70.720787187352826	70.72078719035557
0.90	70.834734336363510	70.834734401933645	70.83473440486307
1.00	70.954954584987661	70.954954661509220	70.95495466601488

## Results and discussion

In this part, we examine the behavior of fractional SIR epidemic model as described in Fig. 1 through GWCM using Caputo fractional derivative. We provide numerical simulations to demonstrate the efficacy of the considered method by considering the initial conditions as $S_0=620$, $I_0=10$ and $R_0=70$. Figure 2 illustrates the behavior of individuals who are suspected to be affected by the disease $S(\tau )$ for diverse fractional orders. We observed that as time increases and $\gamma$ decreases, the number of individuals affected by short-termed influenza epidemic disease decreases. Figure 3 depicts the graphs of behaviour of infected individuals by taking $\gamma =0.3,\ 0.5,\ 0.6,\ 0.7,\ 0.9,\ 1.$ As time $\tau$ increases and fractional order $\gamma$ decreases, the infected people $I(\tau )$ increases. The data demonstrates that our proposed approach is accurate and more efficient. Figure 4 displays how the recovered individuals $R(\tau )$ behave for various fractional order. The number of recovered individuals increases, as time increases and the fractional order $\gamma$ value decreases. Also, we illustrated the numerical values obtained for $\gamma =1$ using suggested method along with RK4 method and RPSM which displayed through graphs in Fig. 5. The numerical solution obtained for infected people from the suggested method with other methods and represented in 2D curves as shown in Fig. 6. The graphical representation of the approximate solutions of recovered individuals compared with other techniques has been portrayed in Fig. 7 and Fig. 8 describes the achieved solutions of SIR epidemic model 5 with respect to time $\tau$ using suggested method at $\gamma =1$. Tables 1, 2 and 3 presents the obtained numerical values for $S(\tau )$, $I(\tau )$ and $R(\tau )$ via projected approach and illustrated approximate solutions of RPSM and RK4 method. It clearly defines that the projected approach provides extremely precise values, requires less computing work and it is free from controlling parameters than existing techniques. Moreover, we observed that the model’s behavior is highly time-dependent and the numerical simulations have done using Mathematica software. Furthermore, our proposed method is highly effective for analyzing wild class of biological and epidemic models which are occurring in real world phenomenon.

## Conclusion

Epidemic models examine the impact of interaction of individuals and population density affect transmission rates. A mathematical model is a crucial tool for explaining the complexities of disease transmission in the real-world. In this study, we explore the fractional SIR epidemiological model as an extension of the SIR model of classical order and analyse their characteristics via GWCM using Caputo fractional derivative. The suggested model’s outcomes for various values of $\gamma$ are displayed through graphs and also carried out numerical simulations using suggested approach which shows the dynamics of the epidemical model. Our proposed technique provides rapid algorithms, extremely precise values, free from controlling parameters and requires less computing work than existing techniques. This analysis explains how the demonstrated approach helps us to study the impact of fractional order and observed that the model’s behavior is highly time-dependent. Moreover, it gives a more realistic way to analyse complex phenomena and aids in understanding the epidemiological models. We conclude that the current research have broadened to encompass epidemiological models that emerge in various scientific domains. For future research, this model can enhance its realism and applicability by utilizing various strategies like vaccination, social distancing, or quarantine measures into the model, one can assess their influence on the spread of disease for further development. Also, we can extend the model by incorporating the real-world data and adjusting the parameters, which helps in validating the assumptions and improve the prediction accuracy.

## Data Availability

The datasets used and/or analysed during the current study available from the corresponding author on reasonable request.
